# Kidney function estimation equations: a narrative review

**DOI:** 10.1007/s11845-025-03874-y

**Published:** 2025-01-28

**Authors:** Nisha Abdul Khader, Veena Ganesh Kamath, Shobha Ullas Kamath, Indu Ramachandra Rao, Attur Ravindra Prabhu

**Affiliations:** 1 Department of Nephrology, Kasturba Medical College, Manipal, Manipal Academy of Higher Education, Manipal, Karnataka India; 2 Department of Community Medicine, Kasturba Medical College, Manipal, Manipal Academy of Higher Education, Manipal, Karnataka India; 3 Department of Biochemistry, Kasturba Medical College, Manipal, Manipal Academy of Higher Education, Manipal, Karnataka India

**Keywords:** Creatinine, Cystatin C, EGFR, Estimated glomerular filtration rate, Estimation equations, Kidney function

## Abstract

Glomerular filtration rate (GFR) as a marker of kidney function is important in health and disease management because decreased kidney function is associated with all-cause and cardiovascular mortality, progression of kidney disease, predisposition to acute kidney injury (AKI), and for drug dosage modification. While measured glomerular filtration rate (mGFR) is acknowledged as the most accurate method for evaluating kidney function, it is at present not feasible to be applied in the clinical arena. Estimated glomerular filtration rate (eGFR) is preferred due to its convenience, cost-effectiveness, and seamless integration into standard clinical practice for kidney function evaluation. The presence of multiple equations for eGFR with applications to differing populations makes their use challenging for clinicians. We reviewed available estimated glomerular filtration rate (GFR) equations and their application in different clinical settings both in normal and chronic kidney disease (CKD) patients. These formulae incorporate serum creatinine and/or serum cystatin C levels and correlate them with measured kidney function. Among the many available equations, the Chronic Kidney Disease Epidemiology Collaboration (CKD-EPI) equation is the most recommended due to its robustness and accuracy across diverse patient populations. Strengths and limitations of different eGFR equations are discussed emphasizing the importance of selecting the appropriate equation based on specific patient demographics and clinical scenarios. There is need for regional validation studies to ensure the global applicability of these equations, considering the variations in population characteristics.

## Introduction

The glomerular filtration rate (GFR) has significant importance in evaluating kidney function, reflecting how efficiently the kidneys filter waste and remove excess fluids from the blood stream [1]. Precise GFR estimation is crucial for clinical decision-making, enabling healthcare professionals to diagnose and monitor kidney disease, adjust medication doses, and evaluate overall kidney function for personalized patient care [2]. While the gold standard for GFR measurement involves using exogenous markers like inulin clearance, these techniques are often impractical in clinical settings due to their complexity [3]. Alternatively, numerous estimation equations have been developed for the assessment of GFR, utilizing readily available patient factors like age, biological sex, race, and serum levels of endogenous filtration markers, thus providing a holistic approach to GFR estimation that accounts for individual variations [3]. Clinical guidelines and regulatory agencies suggest using estimated glomerular filtration rate (eGFR) as a routine assessment for GFR, with measured GFR (mGFR) suggested for more precise evaluation and is recommended as a confirmatory test. The evolution of eGFR equations reflects advancements in our understanding of GFR and its determinants, with each successive equation building upon the knowledge and expertise gained from its predecessors over time [4, 5]. Consequently, more precise equations are formulated, thereby improving accuracy and reducing error margins in GFR estimation. The origin of equations for estimating GFR can be dated back to the early 1950s, with the first equation proposed by Effersoe in 1957 [6]. Subsequently, the field has witnessed the development of over 50 different estimating equations.

Despite improvements, it is crucial to recognize that no single equation is ideal for all situations, and clinicians should be mindful of their limitations.

In this review, we provide a comprehensive overview of each equation, outlining their limitations and implications for clinical practice.

## Methods

We searched published literature from the PubMed database using a combination of the search terms, “Glomerular Filtration Rate”[Mesh], “glomerular filtration rate,” “estimation equation*,” “creatinine-based equation*,” and “cystatin-based equation*” along with the Boolean operators from 15^th^ January 1996 to 25^th^ January 2024. We excluded commentaries and conference abstracts. Alongside, we performed forward and backward citation searches on published original articles and reviews.

### Assessment of GFR

Measuring true GFR directly in humans is not practical [7]. Instead, it is estimated using clearance measurements or serum levels of filtration markers, which consist of exogenous or endogenous solutes mostly cleared by glomerular filtration [8].

Urinary clearance quantifies marker excretion relative to its plasma concentration, while plasma clearance assesses how effectively a marker is cleared from the blood stream over a period, taking into account its plasma concentration. In certain situations, urinary or plasma clearance of the exogenous marker has been used as the gold standard method to measure kidney function.

Inulin, radioactive or non-radioactive iothalamate, iohexol, diethylenetriamine pentaacetate (DTPA), and ethylenediaminetetraacetic acid (EDTA) have been used to measure GFR [9].

However, due to the constraints presented by the complex, time-consuming, and burdensome nature of directly measured GFR, it has become a routine practice in numerous clinical settings to estimate GFR using blood levels of endogenous markers [8]. Serum creatinine (SCr) and serum cystatin C are the most commonly used endogenous filtration markers.

### Creatinine

Creatinine (Cr) has been the predominant biomarker for GFR estimation due to its global availability, standardized assays, and cost-effectiveness [10].

It is a non-protein nitrogenous waste product generated from the metabolism of creatine and creatine phosphate in muscle tissue [11]. Creatine can be obtained directly from the diet, particularly from meat and fish, where it is absorbed in the small intestine and transported to various tissues via the blood stream [12]. Endogenously, creatine is synthesized in the body from the amino acids arginine, glycine, and methionine [12]. In the kidneys, arginine and glycine react to form guanidinoacetate. Guanidinoacetate is then transported to the liver, where it is methylated to form creatine. Creatine synthesized in the liver or obtained from the diet is transported through the blood stream to muscle cells, where about 95% of the body’s creatine is stored. In muscle cells, creatine is phosphorylated to form creatine phosphate (phosphocreatine), which serves as a quick reserve of high-energy phosphates to regenerate ATP during periods of high energy demand. Creatine and creatine phosphate are continually converted to creatinine at a constant rate through a non-enzymatic, spontaneous process, where the phosphate is removed as inorganic phosphate [12].

The bulk of creatinine production takes place in muscle tissue, consequently, an individual’s muscle mass significantly impacts the concentration of SCr [12].

**Fig. 1:**
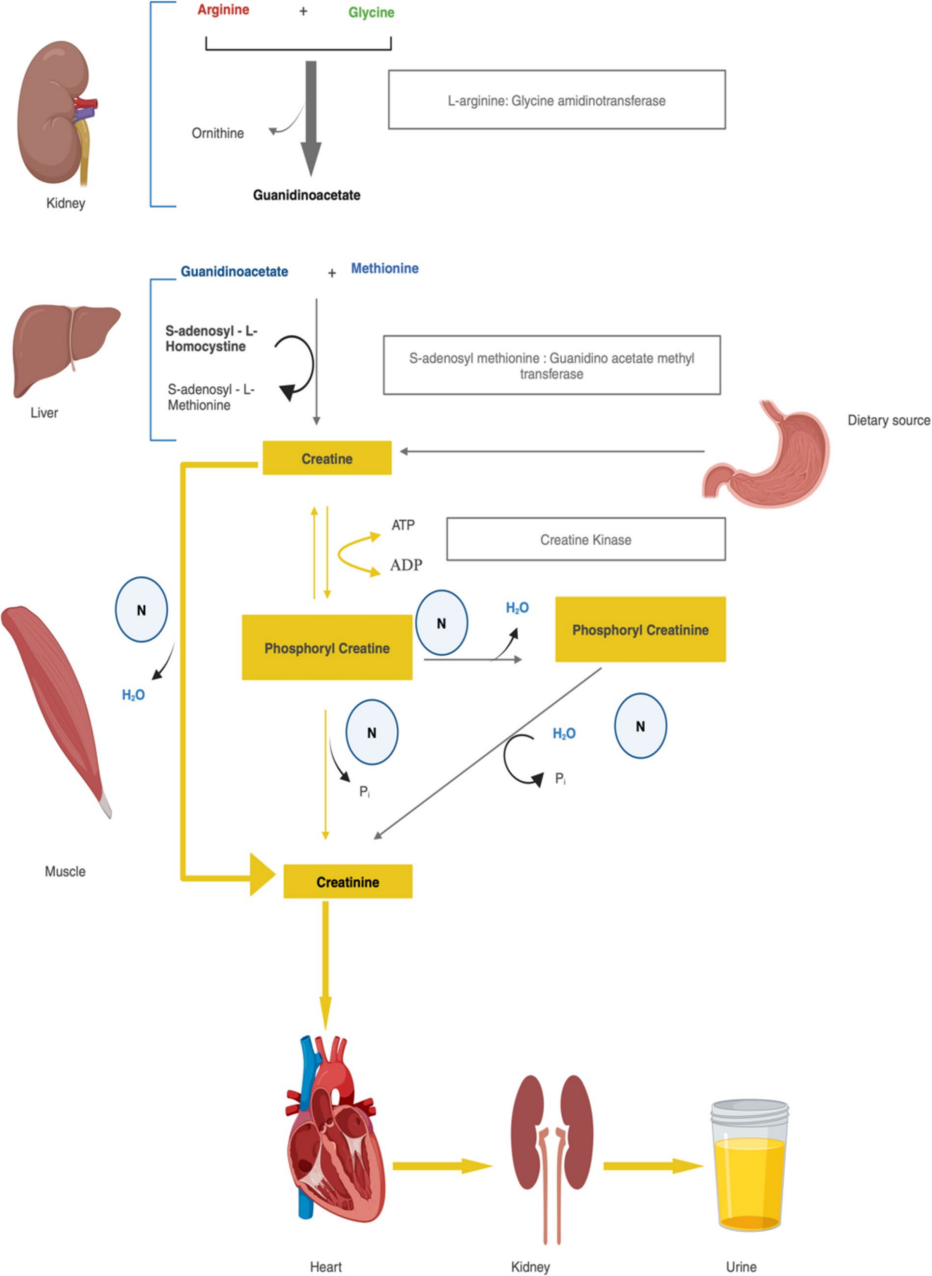
Metabolism of creatinine (created with BioRender.com). H2O, water; N, non enzymatic; Pi, inorganic phosphate

It is consistently released into the blood stream at a steady pace and is cleared by the kidney mainly by glomerular filtration and to some extent by tubular secretion. Increasing tubular secretion with falling GFR keeps SCr levels within range till approximately 50% fall making this not an ideal marker in early CKD [12]. This metabolic pathway is illustrated in Fig. 1.

Mean SCr levels vary between males and females, attributed to variances in muscle mass as well as other non-GFR determinants of SCr [13–15]. The accuracy of eGFR derived from any creatinine-based equations may be less in individuals with significant non-GFR determinants impacting Scr levels. These determinants include variations in muscle mass due to conditions like sarcopenia, cachexia, obesity, malnutrition, and muscular dystrophy, creatine/creatinine intake, specific patient demographics such as children or those with cirrhosis, chronic heart failure, amputations, or neuromuscular diseases, as well as dietary patterns like high-protein or vegetarian diets [16]. In addition, methods for measuring Scr vary and may result in values not being comparable between laboratories. The enzymatic and Jaffe methods represent two colorimetric techniques employed for SCr measurement. Isotope dilution mass spectrometry (IDMS) and high-performance liquid chromatography (HPLC) are utilized as reference standards for SCr measurement. Clinical laboratories commonly employ automated chemical or enzymatic methods for creatinine measurement [16]. The prevalent Jaffe-based creatinine-picrate method, while widely used, is susceptible to interference from factors such as picric acid concentration, pH, wavelength, temperature, and other substances like bilirubin, glucose, protein, aceto-acetate, and cephalosporin. Consequently, this method tends to overestimate creatinine levels by 15–25%. Despite attempts to correct for interference through compensation methods, variability in patient samples limits its effectiveness. Enzymatic methods offer enhanced specificity by preventing interference from non-creatinine compounds but are hindered by higher costs. There is a trend towards normalization of methods to the standard accepted isotope dilution mass spectrometry (IDMS). The National Kidney Disease Education Program (NKDEP) released recommendations for IDMS traceability and standardization of creatinine methods to various stakeholders, including clinical laboratories. IDMS traceability refers to the calibration of the creatinine method utilized (such as enzymatic or the Jaffe method) being traceable to a reference measurement procedure based on IDMS [17–19]. This calibration method ensures accuracy and standardization in creatinine measurements, which is particularly crucial in pediatric populations where creatinine concentrations can vary significantly [20]. Studies have indicated differences in measurements between commercial assays for creatinine, whether Jaffe or enzymatic, underscoring the importance of IDMS traceability to maintain consistency [21]. Implementing IDMS traceable assays can help minimize biases in creatinine measurements, particularly when estimating GFR for clinical purposes, such as in staging chronic kidney disease (CKD).

### Cystatin C

Cystatin C, a member of the cystatin superfamily of cysteine protease inhibitors, is a low-molecular-weight protein that undergoes glomerular filtration without reabsorption but is metabolized in the tubules, precluding direct clearance measurement [22]. Like creatinine, serum cystatin C levels are influenced by various non-GFR factors, such as male sex, greater height and weight, higher lean body mass, increased fat mass, diabetes, elevated inflammatory markers like C-reactive protein, thyroid disorders, and glucocorticoid usage. Increasing age and race also impact cystatin C levels, albeit to a lesser extent than creatinine [23–31]. Hence, GFR estimates incorporating cystatin C should consider these non-GFR determinants.

### Estimation equations for assessing kidney function

To date, there are over 50 different equations in the literature with the most widely used ones being reviewed here.

Estimation equations use mathematical formulas used to evaluate kidney function [32]. In clinical practice, GFR is often estimated using these equations, which include biological biomarkers and demographic variables like sex, age, and sometimes weight, height, and/or ethnicity [33].

The equations convert serum concentration of a filtration marker into an approximation of GFR, often incorporating additional demographic factors. This is particularly relevant as the markers are influenced by these variables.

### Evolution of estimation equations

**Fig. 2:**
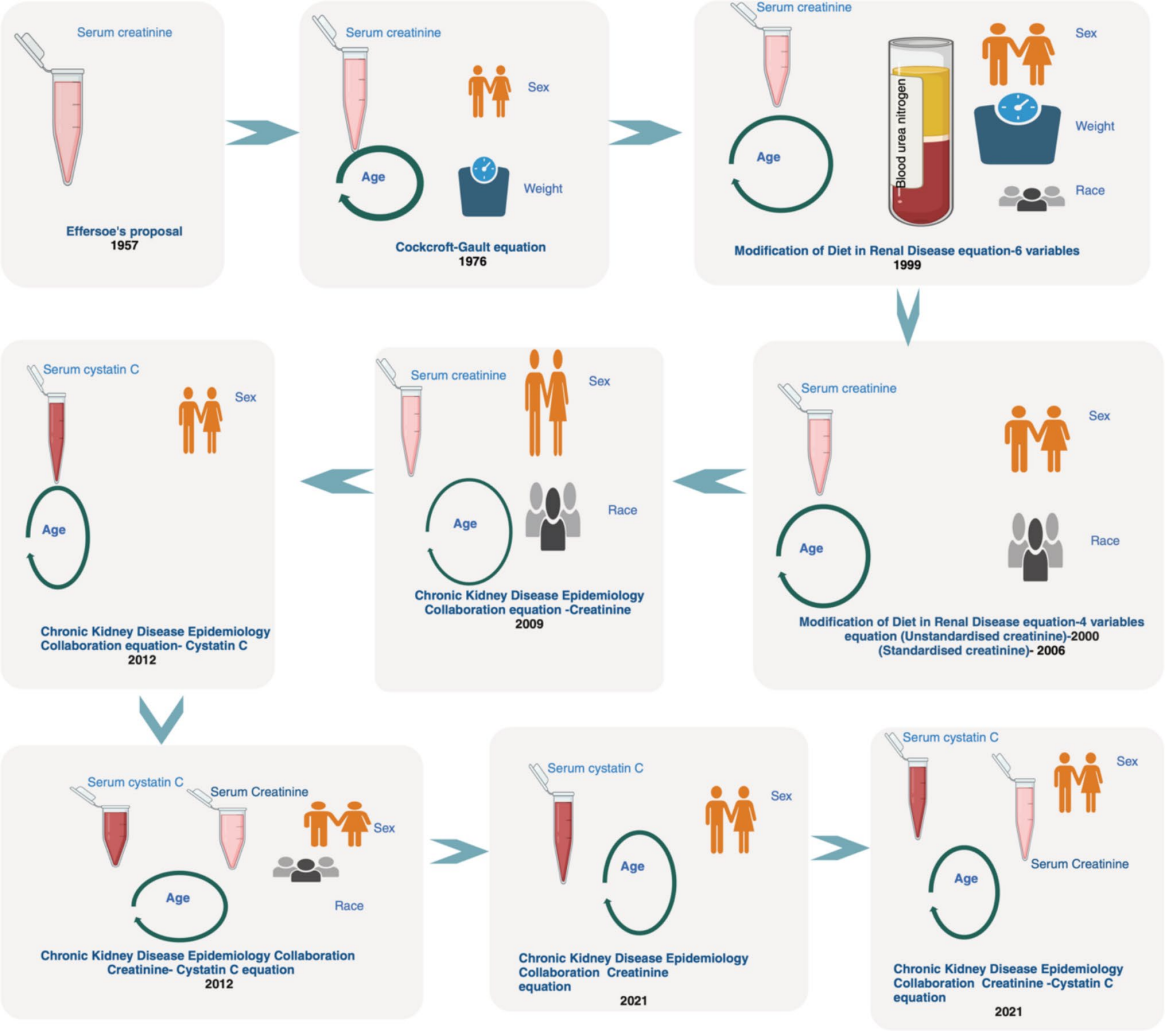
Evolution of estimation equations (created with BioRender.com)

Estimation equations of kidney function have undergone significant evolution over the years marked by significant milestones as depicted in Fig. 2. The initial approaches primarily relied on cumbersome and invasive methods, such as the clearance of exogenous filtration markers [34]. In 1957, Effersoe proposed the first equation for estimating creatinine clearance (CrCl), laying the groundwork for subsequent advancements [6, 35]. In 1976, the introduction of the Cockcroft-Gault equation, based on SCr and patient demographics, marked a significant advancement [36]. Subsequently, the Modification of Diet in Renal Disease (MDRD) equation emerged in 1999, using six variables which was later modified to four variables incorporating SCr, age, ethnicity, and gender. In 2009, the Chronic Kidney Disease Epidemiology Collaboration (CKD-EPI) equation showed improved performance at higher GFR levels, offering enhanced accuracy compared to its predecessor [2]. The Lund-Malmö Study equation, also known as the Lund-Malmö revised (LMR) equation, was developed in 2011 with the explicit goal of improving the accuracy of eGFR. In 2012, the CKD-EPI equation evolved further, integrating cystatin C as an additional filtration marker. The year 2021 saw the advent of a race-free eGFR equation, addressing potential inaccuracies associated with race. The evolution of these equations has shaped the way we estimate GFR, providing more convenient and reliable tools for kidney function assessment.

### Equations for creatinine clearance (CrCl)

#### Jelliffe’s Eq. (1971)

Jelliffe’s equation was developed in 1971 by Dr. Roger W. Jelliffe to estimate CrCl in 15 transplant patients [37–39]. This equation (Table 1) incorporates age, weight, and SCr levels to estimate CrCl. It is a relatively simple formula and has been widely used in clinical practice for decades. Despite its widespread use, this equation has limitations. It was validated to assess CrCl in a non-steady state as in critically ill patients [37]. It may not be as accurate as other equations, particularly in certain patient populations. Additionally, the equation may need to be adjusted for specific clinical conditions or patient characteristics. Adjustment may be necessary for patients with muscle mass significantly above or below normal, such as very muscular patients, cirrhotic patients, those with AIDS, or very obese patients, to prevent overestimation or underestimation of creatinine clearance.

**Table 1:** Equations for estimating CrCL and GFR

Year	Name of the equation	Equation	Population from which the equation is derived	Limitations
1971	Jelliffe	“CrCL = 98 − [0.8 × (age − 20)/serum creatinine × (body mass/1.73) × [0.9 if a woman]”	15 transplant patients	• Development of equation in critically ill patients may restrict its generalizability to other demographics
1974	Kampmann	“CrCL = urine creatinine (mg/kg/min) × weight (kg) × 100/serum creatinine (mg/100 mL)”	368 hospitalized patients without known renal disease	• The equation was derived from hospitalized patients with conditions other than established renal disease
1976	Rowe	“CrCL = 133 − 0.64 × age”	884 adult male community-dwelling subjects	• The equation underwent testing solely in adult healthy males, rendering it unsuitable for application across diverse populations
1976	Cockcroft-gault	“CrCL = (140 − age) × weight (× 0.85 if a woman)/(serum creatinine × 72)”	249 adult hospitalized male patients	• The equation was derived from hospitalized patients, of whom the majority had normal kidney function • Questionable subject selection method (of the 500 male subjects who had 24-h creatinine measurements, only 249 were selected) • Developed without considering modern obesity trends, leading to potential overestimation due to increased fat mass • Lack of adjustment for body surface area may affect accuracy, particularly in individuals with kidney function impairment • Female adjustment factors rely on hypothetical assumptions about muscle mass differences • It was developed before the use of standardized creatinine assays, which resulted in a 10 to 40% overestimation when applied to current laboratory measurements
1976	Schwartz	“GFR = 0.55 × height/serum creatinine”	77 children aged 1 to 21 years	• The Schwartz equation tends to overestimate GFR, especially as renal insufficiency worsens
1987	Keller	“GFR = 130 – age”	368 healthy subjects with normal creatinine	• The equation was tested in a small population with normal creatinine values
1988	Salazar-Corcoran equation	“CrCL (males) = [137-age] × [(0.285 × weight) + (12.1 × height^2^)]/(51 × serum creatinine)” “CrCL (females) = [146-age] × [(0.287 × weight) + (9.74 × height^2^)]/(60 × serum creatinine)”	Animal experiment: Male Sprague–Dawley rats fed either a control diet or an obesity-inducing diet Human sample: 149 males and 219 females of normal stature categorized by age decades	• Further validation of the underlying hypothesis and clinical utility of the proposed equations is necessary through more extensive studies, particularly in patients with moderate to severe renal dysfunction. Additionally, the formulas require evaluation in individuals with normal body weight to ascertain broader applicability
1993	Walser	“GFR = 7.57 × (serum creatinine mmol/L) ^−1^ − 0.103 × age + 0.096 × weight^−6.66”^	85 subjects with advanced CKD aged 23 to 79	• This equation was derived using individuals with advance renal disease. Hence not suitable for those with slightly elevated and normal levels of creatinine • Requires additional measures of urinary urea nitrogen (UNA) for more precise analysis
1995	Nankivell	“GFR = 6.7/serum creatinine (mmol/L) + 0.25 × weight − 0.5 × urea − 0.01 × height^2^ + 35 (25 if a woman)”	146 kidney transplant recipients	• It was designed specifically for estimating GFR in kidney transplant recipients, thus lacking generalizability to other populations
1997	Baracskay	“GFR = 1/2 [100/serum creatinine] + 88 – age”	41 healthy ambulatory geriatric individuals aged 65–85	• Though the equation showed superiority over the CG equation in ambulatory geriatric patients, it still demonstrated significant error (SE = 16.4), casting doubt on its clinical usefulness
1999	MDRD	“GFR (Original MDRD 6 variables) = 170 * (serum creatinine)^−0.999^* (age)^−0.176^ * 0.762 [if female] * 1.180 [if black] * [serum levels of urea (SUNb)]^−0.170^ * [albumin]^+0.318”^	1628 CKD subjects, of both genders who had not undergone dialysis or transplantation and were aged between 18 and 70 years	• The derivation cohort included only CKD patients and exclusion of healthy individuals (selection bias) • Creatinine assays were not calibrated to reference methods • It results in misdiagnosing and misclassifying CKD in individuals with mild renal insufficiency • Use of serum urea nitrogen and albumin
2000		“Simplified MDRD (4 variables) eGFR (mL/min/1.73m^2^) = 186*[serum creatinine]^−1.154^ * [age]^−0.203^ * [0.742 if female] * [1.21 if black]”		• Standardized creatinine was not used • It includes race as a factor
2006		“eGFR using the updated MDRD (4 variables, and standardized creatinine) Eq. (2006) = 175*[serum creatinine]^−1.154^ * [age]^−0.203^ * [0.742 if female] *[1.212 if black]”		• It tends to underestimate the GFR, and it has relatively low accuracy at higher GFR values (above 60 mL/min/1.73m^2^) • Race is a factor
2004	Mayo Quadratic equation	“GFR = exp (1.911 + 5.249/serum creatinine − 2.114/serum creatinine^2^ − 0.00686 *age − 0.205 (if female)) If serum creatinine < 0.8 mg/dL, use 0.8 for serum creatinine”	320 CKD patients and 580 healthy individuals	• The equation was not derived from a sample representing the general population • It does not adequately represent elderly individuals and nonwhite racial groups
2007	Lund-Malmö	**Equation without body weight measure** “e^X−0.0124*age+0.339*In(age)−0.226 (if female)^ X = 4.62–0.0112*plasma creatinine (if plasma creatinine < 150 µmol/L) X = 8.17 + 0.0005* plasma creatinine −1.07*In (plasma creatinine) (if plasma creatinine ≥ 150 µmol/L)” **Equation with lean body mass (LBM)** “e^X−0.00587*age+0.00977*LBM^ X = 4.95–0.0110*plasma creatinine (if plasma creatinine < 150 µmol/L)” “X = 8.58 + 0.0005* plasma creatinine −1.08*In (plasma creatinine) (if plasma creatinine ≥ 150 µmol/L)” **Lund-1 equation without body weight measure** “e^X−0.0168*age+0.523*In(age)−0.208 (if female)^ X = 4.12–0.0111* plasma creatinine (if plasma creatinine < 150 µmol/L) X = 6.51 + 0.0004* plasma creatinine—0.808*In (plasma creatinine) (if plasma creatinine ≥ 150 µmol/L)” **Lund-2 equation with lean body mass (LBM)** “e^X−0.00705*age+0.0110*LBM^ X = 4.93–0.0108* plasma creatinine (if plasma creatinine < 150 µmol/L) X = 7.78 + 0.0005* plasma creatinine −0.902*In (plasma creatinine) (if plasma creatinine ≥ 150 µmol/L)”	850 Swedish Caucasians aged ≥ 18 years	• Uncertainty regarding its applicability in populations with diverse ages, BMI, geographic, racial compositions, and ethnicity • Needs validation in pregnant women, serious comorbidities, and transplant recipients • It tends to underestimate GFR at levels over 90 mL/min/1.73m^2^
2009	Schwartz	“GFR = 0.413 × height/serum creatinine” “GFR = 40.7 × (height/serum creatinine)^0.640^ × (30/Blood urea nitrogen)^0.25”^ “GFR = 39.1 × (height/serum creatinine)^0.516^ × (1.8/cystatin C)^0.294^ × (30/blood urea nitrogen)^0.40^ × (1.099) if male × (height/1.4)^0.9”^	349 children aged 1 to 17 years	• The equation’s derivation from a cohort of 600 children with CKD and abnormal growth limits its accuracy for those with less renal impairment and normal skeletal growth • It overestimates GFR • It relies on the child’s height, which is mostly unavailable in the clinical laboratory setting • Use of blood urea nitrogen
2009	CKD-EPI	“141 × min (serum creatinine/κ, 1)^α^ × max (serum creatinine/κ, 1)^–1.209^ × 0.993^Age^ × 1.018 (if female) × 1.159 (if black), where κ is 0.7 for females and 0.9 for males α is − 0.329 for females and − 0.411 for males min indicates the minimum of SCr/κ or 1 max indicates the maximum of SCr/κ or 1”	8254 subjects from 6 studies and 4 clinical population	• Heterogeneous study pool: studies from different populations were pooled for the equation development and validation, potentially impacting its generalizability • Limited Representation: The study population with higher GFR levels may not fully represent the general population, particularly lacking representation of older individuals and racial minorities • Incomplete data: data on certain clinical factors, such as diabetes type and muscle mass, were incomplete, potentially affecting SCr independently from GFR estimation • Equation complexity: the CKD-EPI equation is more complex than previous equations, although it can be implemented using the same input variables • Creatinine limitations: despite improvements, SCr remains limited as a filtration marker, especially in individuals with abnormal muscle mass levels • Inclusion of race as a factor
2011	Gregori–Macías	“GFR = 2.505458 − (0.264418 × Hematocrit) + (0.118100 × Urea) [+ 1.383960 if male]”	487 subjects with (376) or without (111) chronic renal insufficiency	• This equation may be less accurate in diagnosing CKD in individuals under 70 compared to MDRD and CKD-EPI, as it does not include blood creatinine or CrCL, relying instead on variables like hematocrit, blood urea, and gender • Its accuracy can vary depending on the population studied and may not be universally applicable
2012	CKD-EPI cystatin C	“GFR = 133 × min (serum cystatin C/0.8, 1)^–0.499^ × max (serum cystatin C /0.8, 1)^−1.328^ × 0.996^Age^ × 0.932 (if female), where min indicates the minimum of SCysC/0.8 or 1 max indicates the maximum of SCysC/0.8 or 1”	5352 participants from 13 studies Mean GFR 68 mL/min/1.73 m^2^	• The limitations stem from inconsistencies among commercial assays for Cystatin C, resulting in significant variability in outcomes
2012	CKD-EPI creatinine–cystatin C (2012)	“GFR = 135 × min (serum creatinine/κ, 1)^α^ × max (serum creatinine/κ, 1)^−0.601^ × min (serum cystatin C /0.8,1)^−0.375^ × max (serum cystatin C /0.8, 1)^−0.711^ × 0.995^Age^ × 0.969 (if female) × 1.08 (if black), where α is − 0.248 for females and − 0.207 for males *κ* is 0.7 for females and 0.9 for males min (SCr/κ,1) indicates the minimum of SCr/k or 1 and max (SCr/κ,1) indicates the maximum of SCr/k or 1 min (SCysC/0.8,1) indicates the minimum of SCysC/0.8 or 1 and max (SCysC/0.8,1) indicates the maximum of SCysC/0.8 or 1”	5352 participants from 13 studies Mean GFR 34 mL/min/1.73 m^2^	• Non-availability of cystatin C in all clinical settings • Inclusion of race as a factor
2012	BIS1: creatinine	“GFR = 3736 × creatinine^−0.87^ × age^−0.95^ × 0.82 (if female)”	610 subjects over 70 years of age Mean age: 78.5 years	• Absence of external validation • The BIS equation was specifically developed for older adults, which may limit its applicability and accuracy in younger individuals or those outside the target demographic
2012	BIS2: creatinine and cystatin C	“BIS2 = 767 × cystatin C^−0.98^ × creatinine^−0.77^ × age^–0.94^ × 0.87 (if female)”		• Variability in the measurement of cystatin C levels across different laboratories or assay methods could affect the accuracy and consistency of the equation’s results
2016	FAS	“eGFR = 107.3 × /[serum creatinine/Q] for 2 years < age ≤ 40 years eGFR = 107.3 × 0.988(age − 40)/[serum creatinine/Q] for age > 40 year” where Q is the median SCr	6870 subjects spanning all age groups (< 18 to > 70 years)	• It is anticipated to exhibit better performance in healthy and general populations rather than in patients with CKD
2021	CKD-EPI Creatinine	“GFR = 142 × (Serum creatinine/*A*)* B* × 0.9938age × (1.012 if female) For female, if SCr ≤ 0.7, A = 0.7 and B = − 0.241 and if SCr > 0.7, A = 0.7 and B = − 1.2 For male, if SCr ≤ 0.9, A = 0.9 and B = − 0.302 and if SCr > 0.9, A = 0.9 and B = − 1.2”	8254 subjects 10 studies	• Most data utilized for its formulation originated from the USA, prompting concerns regarding its generalizability to other regions • Estimations of kidney function relying on creatinine derived GFR may exhibit reduced precision in certain demographics, including individuals with diabetes before the development of significant nephropathy, pregnant women, and those with unique body compositions such as obesity, severe malnutrition, amputations, or paraplegia • This method is not suitable for patients undergoing dialysis
2021	CKD-EPI Creatinine-Cystatin C	“GFR = 135 × (Serum creatinine/A)^B^ × (Serum cystatin C /C)^D^ × 0.9961^age^ × (0.963 if female) For female, if SCr ≤ 0.7 and Scys ≤ 0.8, A = 0.7, B = − 0.219, C = 0.8 and D = − 0.323 If SCr > 0.7 and Scys > 0.8, A = 0.7, B = − 0.544, C = 0.8 and B = − 0.778 For male, if SCr ≤ 0.9 and Scys ≤ 0.8 A = 0.9, B = − 0.144, C = 0.8 and D = − 0.323 and if SCr > 0.9 and Scys > 0.8, A = 0.9, B = − 0.544, C − 0.8 and D = − 0.778”	5352 subjects from 13 studies	• Non-availability of cystatin C in all clinical settings

#### Kampmann’s Eq. (1974)

Kampmann and colleagues conducted a study involving 368 hospitalized patients who did not have any known renal disease but had various other medical conditions [40]. These patients were categorized into different age groups based of 10-year spans [40]. The researchers measured 24-h endogenous CrCl, SCr, urinary creatinine excretion, body weight, and height. The researchers measured urinary creatinine in three consecutive 24-h samples and used the mean value as presented in Table 1 [40]. Limitations of this study were that it included only hospitalized patients who did not have a known renal disease, thus restricting the generalizability. The paper does not provide detailed information regarding the specific methods utilized to measure CrCl, SCr, and urinary creatinine excretion. Consequently, the accuracy and reliability of the results may be affected. Additionally, the study does not explore the impact of other factors, such as comorbidities or medication use, on CrCl and SCr levels. Furthermore, the authors emphasize the risk of overestimating GFR when relying solely on SCr as a parameter for assessing renal function.

#### Rowe’s Eq. (1976)

The study aimed to establish age-adjusted standards for CrCl, an important indicator of kidney function. Rowe et al. conducted standard 24-h CrCl measurements on 884 adult male subjects aged 17 to 96 [41]. They were community-dwelling and highly educated without evidence of renal or other diseases. The study found a significant decline in CrCl with advancing age. Using linear regression analysis, a nomogram was developed to determine an individual’s age-adjusted percentile rank in CrCl [41]. The Rowes equation for CrCl in males uses only age as the factor and is depicted in Table 1, whereas for females, 0.93 must be multiplied to the equation for males (Table 1). This equation was solely based on age, which may not accurately reflect the kidney function. Consequently, it is unsuitable for application to all populations, as it was tested solely on healthy adult males.

#### Cockcroft-Gault equation (C-G equation) (1973)

The Cockcroft-Gault (CG) equation was initially developed using data from a homogeneous cohort of 249 adult male patients admitted to a single veterans hospital in Canada, in 1973 [42, 43]. These patients exhibited renal function ranging from normal to mildly or moderately impaired (CrCl: 30–130 mL/m^2^) [42]. Due to the limited diversity within the derivation cohort, the applicability and accuracy of the equation to broader patient populations remain uncertain. As a result, various correction factors have been explored to enhance its utility beyond the original study context. One such correction factor involves a 15% reduction in estimated CrCL for female patients to accommodate differences in muscle mass and fat composition compared to male patients. Additionally, the SCr assay utilized in the derivation of the CG equation differs from contemporary standardized assays, such as isotope dilution mass spectrometry (IDMS)-traceable assays [44, 45]. Unfortunately, the equation cannot be adjusted to account for this change in assay methodology, resulting in an approximate 10% overestimation of CrCl [46]. It estimates CrCl based on age, weight, and SCr levels as described in Table 1. Furthermore, the CG equation incorporates age as a linear function, despite subsequent research indicating non-uniform changes in GFR over time [47].

Despite its historical significance and initial accuracy in diverse patient populations with conditions like hypertension, congestive heart failure, and chronic renal failure, the CG equation has fallen out of favor due to several limitations [48]. These include its reliance on outdated creatinine assays, lack of adjustment for body surface area, and questions surrounding its mathematical validity [43, 49]. Nevertheless, it may still find utility in specific scenarios, such as in elderly patients, for drug studies, and for estimating renal function at the bedside [49]. Its accuracy is compromised in certain populations, such as those with cirrhosis, and it cannot be readily adapted for use with IDMS-traceable SCr values [50, 51].

#### Keller’s Eq. (1987)

The authors described a simple formula designed to estimate CrCl in 25–100 subjects with normal creatinine values [52]. This equation relies solely on one variable, age, and its details are outlined in Table 1. The authors concluded that the equation holds considerable significance in predicting the decline in GFR due to the ageing process among healthy older individuals [52].

#### Salazar-Corcoran Eq. (1988)

In a study, the researchers developed gender-specific equations to estimate CrCl in obese individuals by incorporating fat-free body mass (FFBM) as a key variable [53]. These equations (Table 1) were validated using data from both animal models and human subjects, demonstrating strong correlations between FFBM and CrCl. In contrast to existing methods, these equations accurately predicted CrCl in obese individuals, surpassing the performance of traditional methods [53]. The researchers concluded that these equations provide a reliable tool for optimizing drug dosing in obese patients when direct measurement of CrCl is not feasible [53].

### Estimation equations for GFR

#### Schwartz (1976–2009)

Equations used to estimate GFR in adults are deemed unsuitable for children aged ≤ 9 years. Instead, various equations have been formulated to estimate GFR in them, with the Schwartz formula being the most widely utilized since its inception in 1976 [54]. This equation, represented as eGFR = (*k* × *L*)/SCr, where *k* varies based on the child’s age and *L* denotes length or height, has been found to consistently overestimate GFR. This overestimation is attributed to factors related to the creatinine assay technique and measurement peculiarities specific to children. Notably, the Jaffe method, commonly used for creatinine measurement, may be affected by plasma proteins, leading to an inaccurately high correction factor, particularly in children with lower plasma protein levels. Additionally, due to children’s lower muscle mass, any measurement error has a relatively larger impact compared to adults. To address these concerns, a simplified version of the Schwartz equation, developed in 2009 using standardized creatinine methods, provides a good approximation of the original formula [55, 56]. This simplified equation as given in Table 1 offers a practical alternative and is based on more straightforward inputs. This equation offers a reliable estimate comparable to the more intricate Schwartz eGFR formula, which incorporates variables like creatinine, urea, cystatin C, and height [54]. Cystatin C has been proposed as a potentially superior indicator of renal function in children compared to creatinine [55]. However, it is important to acknowledge a limitation: the equation was developed based on data from a cohort of 600 children with CKD experiencing abnormal growth. Consequently, its accuracy may be compromised when applied to children with milder renal impairment and typical skeletal development.

#### Walser’s Eq. (1993)

Walser et al. observed 85 patients aged 23 to 79 years with advanced CKD (SCr > 2 mg/dL) [57]. They measured SCr and urinary clearance of Technetium-99 m diethylenetriaminepentaacetic acid (mTcDTPA) in these patients. The authors derived the GFR prediction equation from creatinine, age, and weight as given in Table 1 [57]. GFR was expressed per 3m^2^ of height^2^ instead of 1.73 m^2^ of surface area as weight changes, whereas height remains relatively constant [57]. This equation accurately predicted the GFR in the patients, with a small margin of error. However, they are only applicable to patients with SCr levels greater than 2 mg/dL [57].

#### Nankivell’s Eq. (1995)

As existing formulas designed for assessing CKD patients’ kidney function showed inadequate performance when applied to kidney transplant recipients, the authors aimed to investigate the inaccuracies and their underlying causes [58]. Subsequently, they sought to develop specific predictive equations for estimating GFR tailored to renal transplantation. In a cohort comprising kidney transplant recipients (*n* = 146), the study evaluated factors influencing GFR beyond SCr levels [58]. These factors included sex, height, weight, serum urea, duration of dialysis, occurrences of rejection and infections, and prednisolone dosage [58]. The GFR was evaluated in these patients using 9mTe DTPA as a reference method. The analysis revealed a highly variable relationship between SCr and GFR, influenced by factors such as changes in muscle mass and catabolic rate, with additional variations observed in cases of acute tubular necrosis (ATN) and chronic rejection. From this analysis, three alternative GFR formulas were developed and compared against six existing methods of GFR estimation. The newly derived formulas demonstrated the highest correlation, minimal overall bias, the least scatter of the sum of squares, and minimal error, particularly at low GFR levels [58]. Additionally, the study presented two simplified versions of these formulas, requiring fewer clinical parameters for rapid calculation [58]. The association between SCr and GFR exhibits significant variability, influenced by factors such as muscle mass and catabolic rate alterations, potentially impacting the formulas’ accuracy. Additionally, beyond SCr levels, factors like sex, height, body weight, serum urea, dialysis duration, rejection and infection instances, and prednisolone dosage can affect GFR, posing challenges for the derived formulas to fully account for these variables. Clinical conditions like acute tubular necrosis (ATN) and chronic renal rejection may further complicate matters by reducing the tubular secretion of creatinine, not adequately captured by the formulas. Moreover, most formulas tend to systematically overestimate GFR and poorly correlate with the reference method, suggesting limitations in predictive accuracy. Furthermore, formulas developed specifically for kidney transplant recipients may lack generalizability to other patient populations or settings. In extreme cases, such as pediatric patients or adults at the extremes of body weight or height, predictive formulas often lose accuracy, potentially limiting their applicability.

#### Baracskay’s Eq. (1997)

The authors aimed to assess 3 frequently used estimators of GFR (CrCl, CG equation, and 100 divided by SCr) by comparing them to iothalamate clearance (IC) in a cohort of forty one healthy ambulatory geriatric individuals aged 65–85 [59]. IC was observed to decline by 1 mL/min for each year of age in this cohort [59]. While CrCl showed a similar decline and moderate correlation (0.83) with IC, 100/SCr showed a weaker correlation (0.56), substantial positive bias (41 mL/min), and no age-related decline, necessitating an age correction similar to the CG formula. Despite age and weight corrections in the CG formula, its estimates were deemed inaccurate (correlation = 0.5). Consequently, a simpler age-corrected formula (Table 1) was derived, which outperformed the CG equation in this specific geriatric sample but still exhibited significant error, questioning its clinical utility. This underscores the challenges of accurately estimating GFR in elderly populations using SCr-based methods.

#### Modification of diet in renal disease (MDRD study equation) (1999 to 2007)

Levey et al., in the year 1999, first introduced the MDRD study equation. It was derived as a part of the MDRD study in the late 1980s to early 1990s [60]. Initially, the MDRD study aimed to assess the effects of dietary protein restriction and blood pressure control on CKD progression. Consequently, the authors developed six variables equation to estimate GFR using various patient characteristics and laboratory parameters such as SCr, age, gender, ethnicity, blood urea nitrogen (BUN), and serum albumin [60]. The MDRD 6 variables equation demonstrated a high relatedness to GFR (*R*^2^ = 90%) and accuracy within 30% of measured GFR [60].

Later, in 2000, this equation was re-expressed to a simplified equation to enhance its clinical utility, incorporating four variables: age, sex, ethnicity, and SCr levels excluding BUN and albumin. In 2006, this equation was updated (Table 1), after the standardization of SCr assays to Isotope Dilution Mass Spectroscopy (IDMS)-traceable methods [9, 45]. The 4-variable MDRD equation also had a similar performance to the former equation with just patient demographics and SCr [45]. However, this equation is specifically designed for individuals with CKD and has not been thoroughly assessed in individuals with normal kidney function, specific age groups, pregnant women, transplant recipients, or those with comorbidities [60]. Subsequently, the performance of this equation has been evaluated across different demographic groups, as it was initially derived from a cohort of Caucasian Americans. Several adjustments to the equation’s coefficients were made to address differences in body mass and dietary factors among ethnically diverse populations, resulting in varying levels of predictive accuracy. The MDRD equation gained widespread acceptance in regions like the United States, Europe, and Australia, where eGFR is now routinely provided alongside SCr measurements. The primary drawbacks of the MDRD equation include its tendency to underestimate GFR and its reduced accuracy, particularly at higher GFR values. When evaluated against the gold standard of measured GFR (mGFR), the MDRD study equations demonstrated superior performance over the CG equation in CKD patients. The common limitation to both CG and MDRD equations is that these equations rely on steady-state conditions and are not valid in dynamic conditions like AKI [42, 45].

#### Mayo Quadratic Eq. (2004)

In a cross-sectional study, Rule AD et al. formulated an equation based on data from 328 consecutive patients undergoing iothalamate clearance for CKD evaluation and 580 subjects undergoing iothalamate clearance for kidney donor assessment. The equation, detailed in Table 1, was designed to estimate logarithmic GFR using variables including 1/SCr, 1/SCr^2^, age, and sex. Despite outperforming the Cockcroft-Gault and Modification of Diet in Renal Disease (MDRD) equations, this equation was not devised for the general population. Moreover, it inadequately represented African Americans and the elderly. It is predicated on the assumption that individuals with normal SCr values can be likened to a cohort where 14% have CKD and 86% are potential donors, possibly healthier than the general populace. However, these potential donors were not devoid of comorbidities; 29% had hypertension, and 33% had hyperlipidaemia [61].

#### LUND‐MALMÖ Eq. (2007)

Björk et al. drew upon a cohort from Lund (n: 436) to formulate 2 equations for predicting GFR: one based on plasma creatinine, age, and gender (LM), and another with the inclusion of lean body mass (LM_LEM_) [62]. Subsequently, validation of these equations was conducted in a separate sample from Malmö (n: 414). The LM and LM_LEM_ equations exhibited comparable performance in terms of correlation, bias, and accuracy, performing equally well in both men and women. However, the LM_LEM_ equation addressed the significant biases observed in underweight and obese men with the LM and MDRD equations. Although the equations were specifically developed and validated for Swedish Caucasian individuals, their applicability to other ethnic groups remains unclear [62]. These findings were part of a study that evaluated various equations for GFR prediction in adult Swedish Caucasians, comparing them with established equations like MDRD and Mayo Clinic, using plasma creatinine assays. The Lund–Malmö equations, with and without LBM, were found to perform similarly in both derivation and validation samples. Among them, the LM_LBM_ equation exhibited the highest accuracy, significantly outperforming MDRD_IDMS_ [62]. Notably, MDRD_IDMS_ tended to overestimate GFR, particularly in elderly individuals and those with higher BMI. Overall, the Lund–Malmö equations showed superior performance in Swedish Caucasians, with the inclusion of the LBM term improving accuracy in specific subgroups.

#### CKD‐EPI Eq. (2009–2021)

The CKD-EPI consortium introduced a new equation, referred to as the CKD-EPI_2009_ equation, in 2009 [63]. This equation, derived from diverse cohorts including healthy subjects, featured a notably larger sample size for both development (*n* = 8254) and validation datasets (*n* = 3896) compared to the CG and MDRD equations [63]. It utilized mostly urine iothalamate clearances for GFR measurements, with SCr concentrations standardized to IDMS traceability. To address underestimation in healthy individuals, the authors proposed adjusting the serum creatinine exponent based on gender-specific thresholds. Validated with large participant samples, the CKD-EPI equation surpassed the MDRD and CG equations, particularly in GFR estimation. It demonstrated reduced bias, improved precision, and greater accuracy, with a significantly higher percentage of eGFR falling within 30% of measured GFR compared to the MDRD equation [63]. Widely adopted globally, this American equation is presently recommended for adults by the Kidney Disease: Improving Global Outcomes (KDIGO) guidelines. Though it is accepted worldwide, it is not exempt from limitations, particularly in its handling of age and race. The CKD-EPI_2009_ equation, recommended for adults over 18 years old, treats age as a continuous variable, assuming serum creatinine’s relationship with GFR remains constant from 18 years onwards [63]. However, this oversimplification does not accurately reflect GFR changes with age, as GFR typically remains stable until 40 years and declines thereafter. Consequently, the equation tends to overestimate GFR in individuals aged 18 to 30 years [63]. The term “race” is used differently in medical contexts between the United States and other countries. In the U.S., it is often employed due to its inclusion in equations like MDRD and CKD-EPI_2009_, where a “race coefficient” adjusts for potential differences in kidney function among racial or ethnic groups [64]. However, in other countries, the use of “race” in medicine varies. Some emphasize factors like ethnicity or cultural background instead. The coefficient acknowledges the scientific fact that the relationship between SCr and GFR differs between Black or African Americans and non-Black Americans, resulting in higher GFR values in African Americans for the same creatinine concentration [65, 66]. Despite this, the reasons behind this discrepancy remain unclear. While some data suggest a role for muscular mass, there is insufficient evidence to support this claim, and other factors such as creatinine tubular secretion and dietary differences may also play a role [66–68]. Importantly, studies conducted outside the USA have shown that the Black coefficient in the CKD-EPI_2009_ equation does not apply to other Black populations, and the equation performs better without it [68–71]. This underscores the importance of recognizing population-specific differences rather than relying on broad racial categories [71]. Additionally, corrections applied at the GFR level, such as the race coefficient, may be misleading, and adjustments at the creatinine level would be more appropriate [64, 71–73].

The CKD-EPI equations have been developed for cystatin C and a combination of cystatin C and creatinine. These equations, including the CKD-EPI Cystatin C developed in 2012, provide accurate estimations of GFR from serum cystatin C, age, and sex, comparable to the CKD-EPI Creatinine 2009 Equation [74]. Additionally, cystatin C can serve as a valuable confirmatory test for CKD and improve GFR estimation accuracy, especially in patients with muscle wasting or chronic illness. Studies have demonstrated that estimating GFR using both cystatin C and creatinine together is more accurate than using either marker alone [75]. The KDIGO 2024 guidelines recommend initial GFR assessment using SCr and a GFR estimating equation, with confirmatory testing suggested in specific scenarios using serum cystatin C or clearance measurements. Clinical laboratories are advised to use standardized assays for creatinine and cystatin C and report eGFR using the CKD-EPI equations or alternative equations if they demonstrate superior performance [76].

A significant societal push has prompted a recent revision of all the CKD-EPI equation using race as a factor. Since 2019, various arguments have emerged in the USA advocating for the removal of racial factors deemed discriminatory. Notably, researchers highlighted that Black Americans tend to have higher estimated GFR levels compared to non-Black individuals at the same creatinine level, potentially impacting kidney transplant eligibility timelines [67, 77]. In response, the CKD-EPI consortium introduced a new creatinine-based equation, known as the CKD-EPI_creat2021_ equation, which garnered immediate support from prominent medical organizations upon its publication in the New England Journal of Medicine [78]. This race-neutral equation effectively resolved the controversy surrounding racial considerations in the USA. However, the majority of data used for its development were from the USA, raising questions about its applicability elsewhere.

#### Gregori–Macías (2011)

Gregori et al., in their study, aimed to identify a screening test capable of distinguishing between CKD and the natural decline in GFR associated with ageing [79]. Medical data from 487 individuals aged 16–102, obtained from various medical specialists in Argentina, Portugal, and Spain, were analyzed using logistic regression techniques. A formula, termed HUGE, was derived (Table 1). This formula, incorporating hematocrit, blood urea, and gender, accurately diagnoses CKD independent of age, blood creatinine, CrCL, or other eGFR variables. A positive value obtained from the equation indicates the presence of CKD, while a negative value suggests its absence. Their findings indicate that the HUGE formula outperforms traditional methods such as MDRD and CKD-EPI, particularly in individuals over 70 years old [79]. The HUGE screening formula offers a simple, readily available, and cost-effective approach to differentiate between CKD and eGFR < 60 mL/min/1.73 m^2^, potentially preventing a significant number of healthy elderly individuals, estimated at approximately 1.7 million in Spain and 2.6 million in the UK, from being incorrectly excluded from clinical assessments or treatments contraindicated in CKD. This formula, while useful, may exhibit lower accuracy compared to established formulas like MDRD and CKD-EPI when diagnosing CKD in individuals younger than 70 years old. Its reliance on hematocrit, blood urea, and gender as variables may overlook certain factors contributing to CKD, as it does not consider blood creatinine, CrCL, or other eGFR values. Additionally, the formula’s accuracy may be influenced by the specific population under study and may not be universally applicable to all individuals.

#### Berlin Initiative Study (BIS) (2012)

As the accuracy of existing eGFR equations for older individuals (≥ 70) remains uncertain, the authors of the study aimed to develop a new GFR estimator specifically tailored for individuals aged 70 years and above [80]. Conducted as a cross-sectional study, data from 610 participants were utilized, with a division into development and internal validation subsets. Iohexol plasma clearance served as the gold standard for measuring GFR, while the performance of the newly derived BIS equations (BIS1: creatinine-based; BIS2: creatinine- and cystatin C-based) was compared against established estimating equations. Results showed that the BIS2 equation demonstrated the least bias, followed by BIS1 and Cockcroft–Gault equations, with other equations notably overestimating GFR [80]. The BIS equations highlighted a substantial proportion of older individuals with GFR < 60 mL/min per 1.73 m^2^, with the BIS2 equation exhibiting the lowest misclassification rate. While limitations included the absence of external validation, the findings suggest the BIS2 equation as a preferable option for estimating GFR in individuals aged 70 years or older with normal or mildly reduced kidney function, with BIS1 serving as an alternative in the absence of cystatin C data.

#### Full age spectrum Eq. (2016)

Due to variations in the methodologies used to develop equations for different population groups, a discontinuity may arise when transitioning from pediatric to adult equations or from adult to older adult equations. The Full Age Spectrum (FAS) equation, introduced in 2016, aims to address this disparity by formulating a single eGFR equation applicable across all age groups [81]. This is achieved by standardizing SCr for age in children and adolescents and for sex in adolescents and adults, using a normalized SCr (SCr/Q) approach, where Q represents the median SCr from healthy populations, adjusted for age and gender. Validation of the equation involved 6870 individuals, encompassing children, adults, and older adults, with measured GFR (using inulin, iohexol, and iothalamate clearance) and IDMS-equivalent SCr. Bias, precision, and accuracy were evaluated, revealing that the FAS equation demonstrated less bias and greater accuracy compared to the Schwartz equation in children and adolescents and comparable performance to the CKD-EPI equation in young and middle-aged adults. Additionally, it exhibited superior accuracy and less bias than the CKD-EPI equation in older adults. While further validation may be required in diverse ethnic populations and for the derivation of *Q* values, external validation suggested that the FAS equation may outperform the Schwartz and CKD-EPI equations, particularly for older adults with mGFR > 60 mL/min/1.73 m^2^. Although the FAS equation is based on SCr/Q values for a healthy population, it is anticipated to perform well in a healthy and general population, with comparable performance to the CKD-EPI equation in subgroups with mGFR < 60 mL/min/1.73 m^2^ during validation [81].

### Limitations of estimation equation

Though there are many equations to estimate eGFR, not all equations are suitable for estimation across different demographics. Estimating equations for GFR have limitations because they only represent average relationships between the marker and its non-GFR determinants. Understanding this is crucial for accurately assessing GFR in various individuals and clinical scenarios [82]. The majority of estimation equations are based on creatinine levels and some on cystatin C. The limitations of using creatinine and cystatin C to estimate GFR are multifaceted:

1. Variations in creatinine production: creatinine production varies among individuals due to factors like diet, muscle mass changes (e.g., amputation, malnutrition), and dietary supplements. The accuracy of estimation equations is affected, especially among lower extremity amputees, due to significant muscle mass reduction [83–85].

2. Variations in creatinine secretion: as GFR declines, the increase in SCr is somewhat counterbalanced by increased secretion of creatinine in the proximal tubules. This can lead to relatively stable SCr levels despite declining GFR, particularly in early kidney disease stages [86–91].

3. Extrarenal creatinine excretion: in advanced kidney failure, extrarenal creatinine elimination increases due to intestinal bacterial overgrowth and bacterial creatinase activity. Consequently, SCr concentration may be lower than expected [92].

4. Requirement for stable SCr: accurate eGFR estimation relies on stable SCr levels. In conditions like AKI, where GFR decreases rapidly, serum creatinine may not reflect the severity of kidney disease until sufficient time has passed for the filtration marker to accumulate [24].

5. Measurement issues: serum creatinine measurement methods and equipment variations can lead to differences in reported values, affecting GFR estimation accuracy. Interfering substances and drugs, as well as differences in assay methods can also impact SCr levels [91, 93–95]. Understanding these limitations is crucial for interpreting eGFR accurately across diverse individuals and clinical scenarios.

6. Non-GFR factors of cystatin C: the levels of cystatin C are influenced by factors such as steroid use, thyroid dysfunction, obesity, and inflammation [96].

7. Cost of cystatin C measurement: measuring cystatin C is ten times more expensive than measuring creatinine [96].

### Limitation of the review

The primary limitation of this review is the existence of over 50 different equations for estimating kidney function, making it impractical to cover each one comprehensively. Consequently, the review focuses on the most widely used equations, potentially overlooking some of the less commonly used ones.

## Conclusion

While numerous equations exist for estimating GFR, none are universally applicable across all patient demographics. The variability in certain factors limits the accuracy of these equations. While no universal consensus exists on the optimal formula for estimating glomerular filtration rate (GFR), the CKD-EPI race-free equation is widely used in clinical practice. Clinicians are encouraged to use this equation for GFR estimation, considering patient-specific factors. The development of equations tailored to specific populations, validated across diverse groups, and adjusted for individual patient characteristics, is essential for improving the precision of GFR estimations in clinical practice.

## Future prospects and recommendations

This review highlights the importance of developing new equations that include novel endogenous filtration markers beyond creatinine and cystatin C to improve precision and accuracy in a cost-effective manner. It recommends validating these equations across various patient populations, including those with different racial backgrounds, ages, and health conditions, to ensure broader applicability and accuracy. There is a need to integrate personalized medicine approaches by considering individual patient characteristics. It highlights the importance of leveraging advanced computational methods and large datasets to refine existing equations and develop novel predictive models. Future research efforts should concentrate on overcoming challenges to make directly measured GFR feasible in clinical settings, given its superior accuracy and precision in assessing kidney function. Additionally, there is a need for advancements in point-of-care testing for kidney function.
